# Heterogenous biofilm mass-transport model replicates periphery sequestration of antibiotics in *Pseudomonas aeruginosa* PAO1 microcolonies

**DOI:** 10.1073/pnas.2312995120

**Published:** 2023-11-13

**Authors:** Joshua Prince, A-Andrew D. Jones

**Affiliations:** ^a^Department of Civil and Environmental Engineering, Duke University, Durham, NC 27708; ^b^Thomas Lord Department of Mechanical Engineering and Materials Science, Duke University, Durham, NC 27708

**Keywords:** antimicrobial resistance, *Pseudomonas aeruginosa*, biological barriers, biofilm transport, cystic fibrosis

## Abstract

A model for antibiotic accumulation in bacterial biofilm microcolonies utilizing heterogenous porosity and attachment site profiles replicated the periphery sequestration reported in prior experimental studies on *Pseudomonas aeruginosa PAO1* biofilm cell clusters. These *P. aeruginosa* cell clusters are in vitro models of the chronic *P. aeruginosa* infections in cystic fibrosis patients which display recalcitrance to antibiotic treatments, leading to exacerbated morbidity and mortality. This resistance has been partially attributed to periphery sequestration, where antibiotics fail to penetrate biofilm cell clusters. The physical phenomena driving this periphery sequestration have not been definitively established. This paper introduces mathematical models to account for two proposed physical phenomena driving periphery sequestration: biofilm matrix attachment and volume-exclusion due to variable biofilm porosity. An antibiotic accumulation model which incorporated these phenomena better fit observed periphery sequestration data compared to previous models.

Cystic fibrosis is a genetic disorder mainly caused by mutation in the gene for the cystic fibrosis transmembrane conductance regulator (CFTR) affecting 100,000 worldwide (1). While the drug class known as CFTR-modulators have significantly improved outcomes for people with cystic fibrosis, patients still acquire chronic *Pseudomonas aeruginosa* infections (1). In many cases of chronic *P. aeruginosa* lung infections, the bacteria have formed a biofilm that is recalcitrant to common antibiotic therapies (2). Two classes of hypothesis have formed to explain this antibiotic-biofilm recalcitrance: antibiotic diffusion-limitations and physiology-based mechanisms (3). The diffusion-limitations hypothesis states that specific or nonspecific physical interactions between an antibiotic and the biofilm extra-cellular matrix lead to slower antibiotic penetration into biofilms, preventing antibiotics from reaching and killing interior cells (4). Mathematical models based on this hypothesis quantify this diffusion-limitation by assuming a biofilm with homogenous porosity and antibiotic attachment site density and incorporate antibiotic–matrix interactions into a “homogenized” diffusivity (5, 6). Experiments however have indicated that for certain antibiotics, antibiotic–matrix interactions cause biofilm recalcitrance not due to hindered diffusion but spatial heterogeneity in the biofilm causing antibiotics to accumulate at the periphery of the biofilm termed “periphery sequestration” (7, 8). To account for this periphery sequestration theory, we developed a mathematical model relaxing either or both the assumptions of homogenous biofilm porosity and antibiotic attachment site concentration used in prior models (9, 10). Only the mathematical model which accounted for heterogeneities in both antibiotic attachment sites and porosity replicated both the periphery sequestration of tobramycin and overall antibiotic accumulation of ciprofloxacin in *P. aeruginosa* cell-clusters determined experimentally by Tseng et al. (8). These new models can be applied in designing new antibiotics for chronic *P. aeruginosa* infections for people with cystic fibrosis to circumvent the periphery sequestration mechanism of biofilm-recalcitrance. Further, the methods developed here to capture heterogeneities in both attachment sites and porosity could be applied to transport limitations through other biological hydrogels (4).

## Theory

Antibiotic accumulation into bacterial biofilm microcolonies was modelled using a diffusion-reaction mass-transport approach (11, 12). The biofilm was approximated as a thin-film of height $H\;\left[m\right]$ along a single depth-coordinate axis, x . The system boundaries were a fluid–biofilm interface and a solid–biofilm interface, both normal to the depth axis of the biofilm and parallel to each other. The liquid–biofilm interface was a constant source of antibiotics, $c_{0,I}\;\left[\text{kg}/m^{3}\right]$ . The solid-biofilm interface was an antibiotic-impermeable substratum, defined as the origin of the one-coordinate system. Antibiotic molecules in the biofilm were defined as either in the biofilm pore space and mobile, $m_{M}\;\left[\text{kg}\right]$ , or attached to biofilm biomass, $m_{A}\;\left[\text{kg}\right]$ . Antibiotic concentrations were defined on either a total biofilm volume basis, $c_{M,T}=m_{M}/V_{T}\left[\text{kg}/m^{3}\right]$ , or a biofilm interstitial-volume basis, $c_{M,I}=m_{M}/V_{I}\left[\text{kg}/m^{3}\right]$ . The antibiotic was assumed to be cell impermeable. Biofilm porosity was defined as the ratio of interstitial biofilm volume to total biofilm volume, $\varphi =V_{I}/V_{T}$ , and was used to relate the two concentration definitions, as $c_{M,T}=\varphi c_{M,I}$ and $c_{A,T}=\varphi c_{A,I}$ . The dimensionless mass-balances on mobile, ${\hat{c}}_{M}=c_{M,I}/c_{0,I}$ , and attached antibiotic, ${\hat{c}}_{A}=c_{A,I}/c_{0,I}$ , respectively, led to the Fick-Jacobs expression (13) for diffusion in systems with varying cross-section with an assumption of reversible antibiotic attachment:

[1] $$\frac{\partial {\hat{c}}_{M}}{\partial \hat{t}}=\gamma \left[\frac{\partial^{2}{\hat{c}}_{M}}{\partial {\hat{x}}^{2}}+\frac{\varphi^{\prime }\left(X\right)}{\varphi \left(x\right)}\frac{\partial {\hat{C}}_{M}}{\partial \hat{x}}\right]-\alpha \left[{\hat{c}}_{M}\left({\hat{c}}_{S}\left(x\right)-{\hat{c}}_{A}\right)-\kappa {\hat{c}}_{A}\right],$$

[2] $$\frac{\partial {\hat{c}}_{A}}{\partial \hat{t}}=\alpha \left[{\hat{c}}_{M}\left({\hat{c}}_{S}\left(x\right)-{\hat{c}}_{A}\right)-\kappa {\hat{c}}_{A}\right].$$

Dimensionless time, $\hat{t}=tD_{W}/H^{2}$ , and position, $\hat{x}=x/H$ , were used to nondimensionalize, where $D_{W}\;\left[m^{2}/s\right]$ is the antibiotic diffusivity in water. The dimensionless groups which arose from this nondimensionalization included $\gamma =D_{B}/D_{W}$ , the effective diffusivity of the antibiotic in the biofilm; $\kappa =k_{M,I}/k_{A,I}c_{0,I}$ , the dimensionless attachment equilibrium constant, where $k_{A/M,I}\left[m^{3}/\left(\mathrm{kg}*s\right)\&s^{-1}\right]$ are the rate of antibiotic attachment/detachment; and $\alpha =k_{A,I}c_{0,I}H^{2}/D_{W}$ , the dimensionless ratio of antibiotic attachment rate to diffusion. The variable cross-sectional area available for diffusion from the Fick-Jacobs expression is accounted for by the porosity factors in the diffusion term. Differentiating this model from previous homogenous biofilm models, attachment site concentration, ${\hat{c}}_{S}\left(x\right)$ , and porosity, φ(x) , were spatially heterogenous. The dimensionless attachment site concentration, ${\hat{c}}_{S}=c_{S,I}/c_{0,I}$ , profile took the form ${\hat{c}}_{S}=\xi {\left(\hat{x}\right)}^{a}+\eta$ , where $\xi =\left(c_{S,LI}-c_{S,SI}\right)/c_{0,I}$ was the dimensionless attachment site heterogeneity constant, $\eta =c_{S,SI}/c_{0,I}$ was the dimensionless solid-interface attachment site concentration, $c_{S,LI}\left[\text{sites}/m^{3}\right]$ was the liquid–biofilm interface attachment site concentration, $c_{S,SI}\left[\text{sites}/m^{3}\right]$ was the solid–biofilm interface attachment site concentration, and a was a profile shape parameter. The porosity profile took the form $\varphi =\varphi_{\mathrm{SI}}-\beta \left(e^{b\hat{x}}-1\right)$ , where $\varphi_{\mathrm{SI}}$ was the porosity at the solid–biofilm interface, $\beta =\left(\varphi_{\mathrm{SI}}-\varphi_{\mathrm{LI}}\right)/\left(e^{b}-1\right)$ was the porosity heterogeneity constant, $\varphi_{\mathrm{LI}}$ was the porosity at the liquid–biofilm interface, and b was a profile shape parameter. These attachment site and porosity profiles are not assumed to be universal across antibiotic-bacterial systems (14). Spatial concentration profiles for ${\hat{c}}_{M}$ and ${\hat{c}}_{A}$ calculated by solving Eqs. **1** and **2** using a set of dimensionless parameter values were translated to total concentration profiles on a total biofilm-volume basis using the definition ${\hat{c}}_{T,T}=\varphi \left({\hat{c}}_{M,I}+{\hat{c}}_{A,I}\right)$ . To quantify the degree of antibiotic sequestration with the biofilm, a “degree of sequestration” quantity, $Se=\left({\bar{c}}_{LI-\delta ,T}-{\bar{c}}_{SI+\delta ,T}\right)/{\bar{c}}_{T,T}$ , was defined where ${\bar{c}}_{LI/SI\pm \delta ,T}$ was the equilibrium average total dimensionless concentration on a total volume basis within 5% of the liquid/solid interface, respectively.

## Results and Discussion

**Fig. 1.**
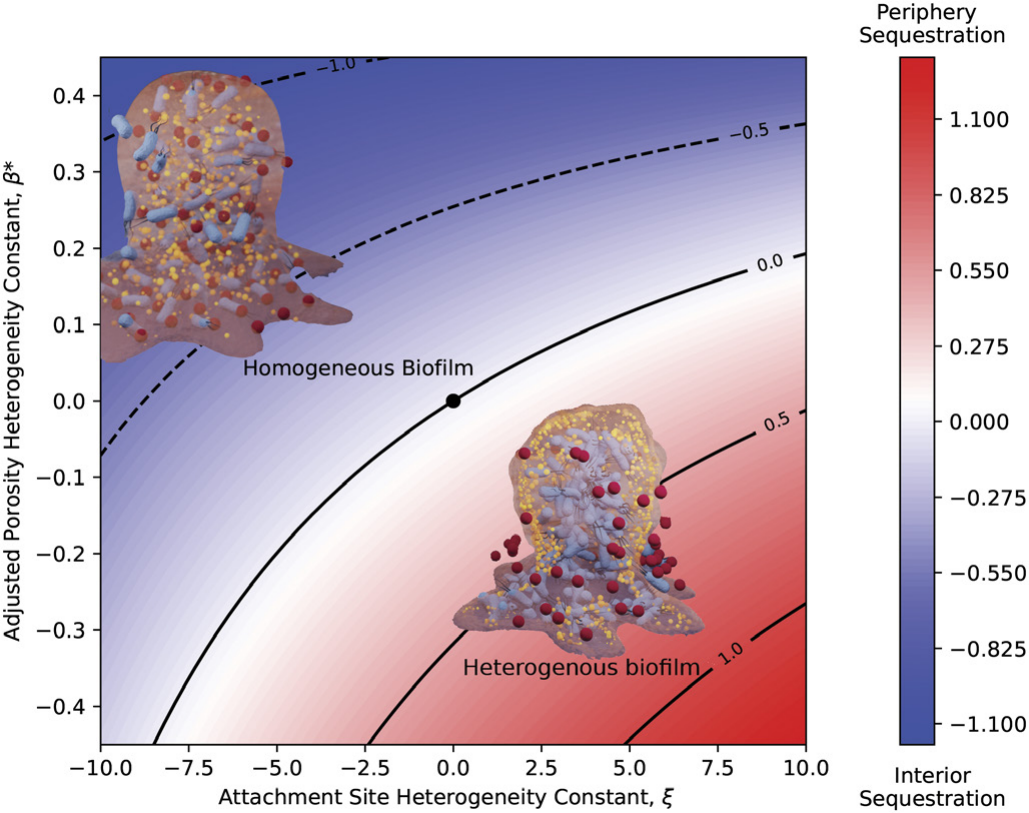
Phase-space plot showing effect of adjusted porosity and attachment site heterogeneity constants ( $\beta^{*}$ and ξ , respectively) on degree of antibiotic sequestration within the biofilm, *Se*. Positive *Se* correspond to periphery sequestration (red), negative *Se* correspond to interior sequestration (blue). Parameter values of γ=1, α=1 , b=5 , a=5 , η=15 , φSI = 0.5, and κ=1 used for solving Eqs. **1** and **2** with given $\beta^{*}$ and ξ values.

This proposed heterogenous biofilm model was tested for its ability to predict periphery sequestration of antibiotic within the biofilm at equilibrium. The effects of the two biofilm heterogeneity constants, $\xi \in \left[-10,10\right]$ and $\beta^{*}=\varphi_{\mathrm{SI}}-\varphi_{\mathrm{LI}}\in \left[-0.5,0.5\right]$ , on Se show that positive ξ and negative β were associated with periphery sequestration and vice-versa for interior sequestration (Fig. 1). A positive ξ corresponded to more antibiotic attachment sites near the periphery to sequester antibiotic compared to the interior. This highlights the divergence of this model from the Crank assumptions for antibiotic-biofilm accumulation and allows for a depth-dependent attachment site concentration site profile (4). A negative β leading to periphery sequestration corresponded to more pore space available for the antibiotic to occupy near the biofilm periphery compared to the interior. This highlights the models ability to account for a “volume-exclusion” effect of porosity where antibiotic is unable to occupy cell-volume, distinguishing it from the Hinson and Kocher method of porosity incorporation, which only modifies the diffusion constant (10). Both the homogenous biofilm model (9) and the homogenized biofilm model (5) can be represented as the origin of the investigated phase-space and shows no degree of sequestration, highlighting their inability to predict periphery sequestration at equilibrium.

**Fig. 2.**
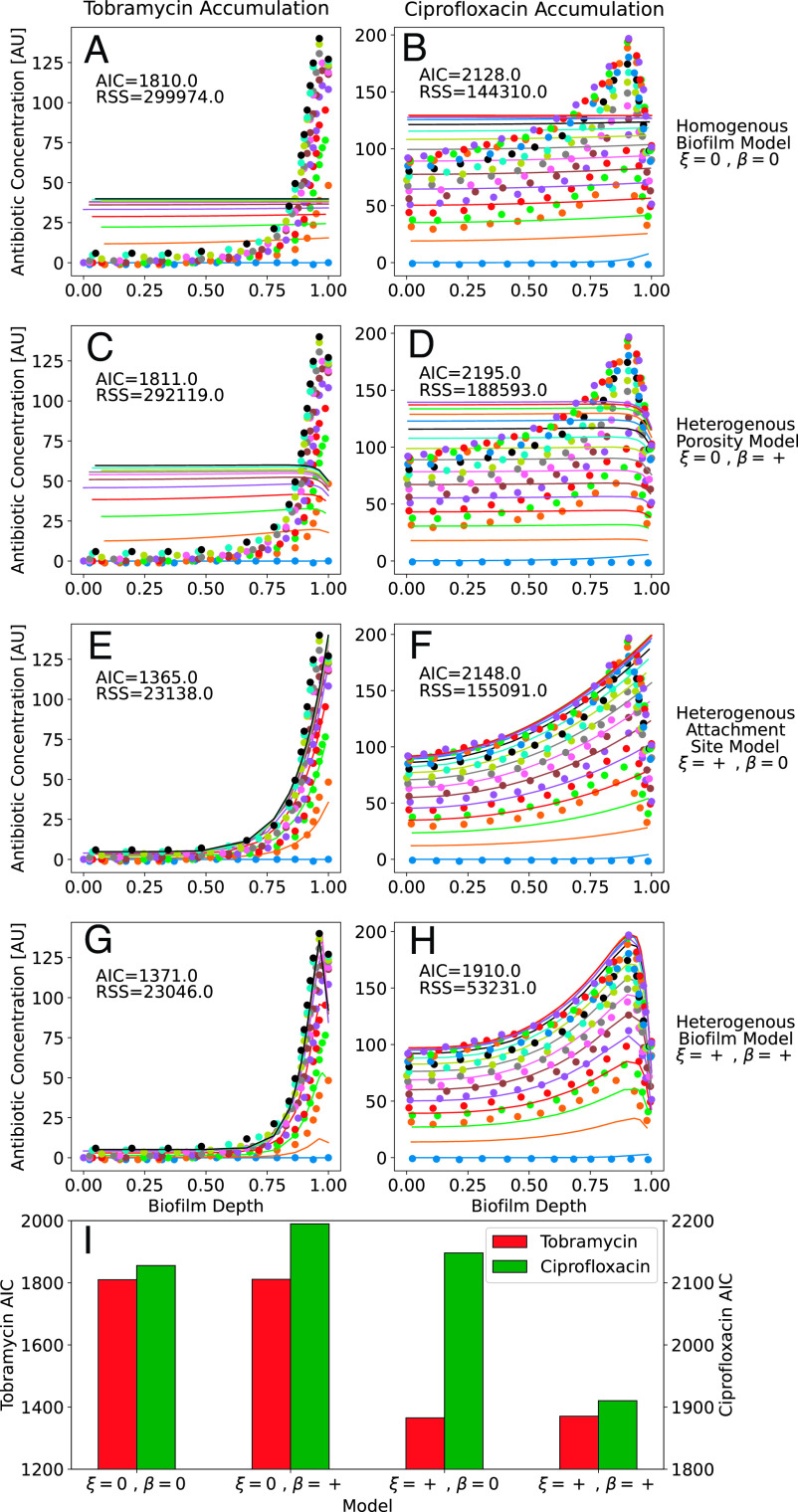
Fits of homogenous biofilm model (*A* and *B*), heterogenous porosity model (*C* and *D*), heterogenous attachment site model (*E* and *F*), and full heterogenous biofilm model (*G* and *H*) to data on accumulation of tobramycin (*A*, *C*, *E*, and *G*) and ciprofloxacin (*B*, *D*, *F*, and *H*) into *P. aeruginosa* PAO1 microcolonies. Dots represent accumulation data from Fig. 2 of Tseng et al. with different colors representing different time-points. Solid lines represent fits of the data using the respective model, with color matched between literature data and model fits. An assumption of negative β   for the porosity profile was inferred from biofilm cross-sections where antibiotic accumulation was tracked. (*I*) Comparison of AIC goodness-of-fit criteria of each model to the tobramycin and ciprofloxacin datasets. Best-fit parameter values for *A*–*H*, respectively: *α* = 1, 0.05, 0.01, 0.01, 0.3, 0.1, 0.2, 0.1; *κ* = 5, 5, 1, 1, 3, 5, 0.001, 20; β^*^ = ø, ø, –0.02, –0.02, ø, ø, –0.55; *b* = ø, ø, 30, 30, ø, ø, 30, 30; *φ_SI_* = ø, ø, 0.1, 0.1, ø, ø, 0.25, 0.25; *ξ* = ø, ø, ø, ø, 150, 10, 250, 35; *a* = ø, ø, ø, ø, 7.5, 2, 14, 3; *η* = ø, ø, ø, ø, 1, 3, 1, 2.5.  All fits used γ=1.

The proposed heterogenous biofilm model replicates the periphery sequestration of tobramycin and overall accumulation of ciprofloxacin in *P. aeruginosa* PAO1 biofilm microcolonies dynamically, with experimental data acquired from Tseng et al. (Fig. 2). Two goodness-of-fit criteria to quantify model fit, the **R**esidual **S**um of **S**quares, $RSS=\sum_{i}y_{O,i}-y_{P,i}$   and the **A**kaike **I**nformation **C**riteria, $AIC=2p+N\left[ln\left(2\pi \sum_{i}RSS/N\right)+1\right]$   , where p   is the number of model parameters and N   is the number of datapoints, were calculated for each model fit to the data. Fits were determined by systematically varying parameter values to find local RSS minimums for each parameter manually. The use of the homogenous biofilm and heterogeneous porosity profile models led to a poor fit to the observed accumulation data for both antibiotics (Fig. 2 *A*–*D*). The heterogenous attachment site profile with a homogenous porosity profile replicated the tobramycin accumulation data (Fig. 2*E*) but had poor goodness-of-fit measures to the ciprofloxacin data (Fig. 2*F*). The model with both a heterogeneous porosity profile and heterogenous attachment site profile (Fig. 2 *G* and *H*) was able to replicate both the tobramycin and ciprofloxacin accumulation, with the AIC goodness-of-fit criteria low or lower than all other models for both antibiotics (Fig. 2*I*). The similarity in performance of models with and without heterogenous porosity for the tobramycin data represented by Fig. 2 *E* and *G* can be attributed to the high density of tobramycin attachment sites, ξ=250   , (2, 7, 8) increasing the effect of attachment site heterogeneity over porosity heterogeneity. Conversely, ciprofloxacin required a balancing of both heterogeneities due to lower attachment site density, ξ=10 , requiring both contributions be included in the model (Fig. 2*H*). In cases of very similar metrics, researchers using these models should experimentally evaluate the relative importance of these heterogeneity mechanisms in relation to model predictions in addition to RSS and AIC. Concentrations at early times (see orange lines in Fig. 2 *F*–*H*) are likely underestimated due to implementation of the proposed model in Cartesian coordinates rather than the cylindrical coordinates more appropriate for this experimental system. Future work could implement algorithmic RSS-minimization techniques for model fits to ensure global minimums. Future work could spatiotemporally measure antibiotic accumulation in biofilm microcolonies concurrently with biofilm porosity profiles and test whether the latter improves predictions of the former using the proposed model.

## Methods

Information on equation solver can be found in the *SI Appendix, Extended Methods*.

## Supplementary Material

Appendix 01 (PDF)

## Data Availability

All study data are included in the main text or associated repository (15).
