# Supplementary material for: Heterogenous biofilm mass-transport model replicates periphery sequestration of antibiotics in Pseudomonas aeruginosa PAO1 microcolonies
Source: Proc Natl Acad Sci U S A. 2023 Nov 13;120(47):e2312995120. doi: 10.1073/pnas.2312995120 (PMC10666002; doi:10.1073/pnas.2312995120)
Supplement: Supplementary file 1 — Appendix 01 (PDF) [file pnas.2312995120.sapp.pdf]

Supplementary Information:

Extended Methods Section

To solve the governing equations used in this paper, a finite-differences solver based on spatial discretization, Newton-Raphson technique, and method-of-lines technique was implemented in Python, with code available on associated repository (1). A variable liquid-interface concentration boundary replaced the constant concentration boundary condition for this dynamic solver due to the dynamic boundary condition in the literature data. Relative antibiotic concentrations were inferred from Tseng *et al* using WebPlotDigitizer, with data available in associated repository (1, 2).

References

1. J. Prince, A.-A. D. J. III, Data from: Heterogenous biofilm mass-transport model replicates periphery sequestration of antibiotics in Pseudomonas aeruginosa PAO1 microcolonies. Duke Research Data Repository.
2. B. S. Tseng *et al.*, The extracellular matrix protects Pseudomonas aeruginosa biofilms by limiting the penetration of tobramycin. *Environ Microbiol* **15**, 2865-2878 (2013).
